# High fat feeding promotes obesity and renal inflammation and protects against post cardiopulmonary bypass acute kidney injury in swine

**DOI:** 10.1186/cc13092

**Published:** 2013-10-31

**Authors:** Philippa Sleeman, Nishith N Patel, Hua Lin, Graham J Walkden, Paramita Ray, Gavin I Welsh, Simon C Satchell, Gavin J Murphy

**Affiliations:** 1 Bristol Heart Institute, University of Bristol, Bristol Royal Infirmary, Bristol BS2 8HW, UK; 2 Department of Anaesthesia and Critical Care, University Hospital Leicester, Infirmary Square, Leicester LE1 5WW, UK; 3 Academic Renal Unit, University of Bristol, Southmead Hospital, Bristol BS10 5NB, UK; 4 Department of Cardiovascular Sciences, University of Leicester, Glenfield Hospital, Leicester LE3 9QP, UK

## Abstract

**Introduction:**

Obesity confers a survival advantage in the critically ill and in patients undergoing cardiac surgery. We explored whether an obesogenic high fat diet could confer protection against post cardiopulmonary bypass (CPB) acute kidney injury (AKI) in a swine model.

**Methods:**

In this study, 28 anaesthetised adult female Landrace White swine (55 to 70 kg) were allocated into a 4 group design to either 2.5 hours of CPB or Sham operation with or without pre-procedural high fat (HF) feeding containing 15% lard, 1.5% cholesterol and 1% cholic acid for 12-weeks (Groups: Sham, CPB, CPB + HF and Sham + HF). Our primary endpoint was creatinine clearance measured at 1.5 and 24 hours post intervention. This is a validated index of the glomerular filtration rate (GFR) in swine and an endpoint used in our clinical studies. Secondary endpoints included measures of systemic and renal inflammation, endothelial homeostasis, tubular injury and dysfunction, and inflammatory cell signalling. Differences between groups were calculated using analysis of variance with adjustment for baseline differences for repeated measures.

**Results:**

CPB in pigs fed a normal chow diet resulted in AKI. This was characterised by reductions in GFR sustained for up to 24 hours post injury relative to Sham operated pigs fed a normal diet; mean difference 50.2 ml/min (95% CI 5.9 to 94.4). Post CPB AKI was also characterised by renal inflammation, parallel activation of both pro-inflammatory (NF-kB, iNOS) and pro-survival pathways (pAkt, p70s6k, HIF-1α) and apoptosis. Pigs fed a 12-week high fat diet developed obesity and hyperlipidaemia. This was associated with increased redox sensitive pro-inflammatory and anti-apoptotic signalling, and tubular epithelial cell proliferation. High fat feeding also protected swine against post CPB AKI; mean difference in creatinine clearance CPB - CPB + HF −65.3 ml/min (95% CI −106.9 to −23.7), by preserving endothelial homeostasis and function, and preventing the reductions in GFR, loss of ATP and tubular apoptosis that characterise the extension phase of AKI in swine at 24 hours post injury. Reno-protection was not attributed to pAkt signaling.

**Conclusions:**

A high fat diet promoted obesity and renal inflammation and prevented post CPB AKI in swine. This study provides insights into the obesity paradox and the failure of anti-inflammatory interventions to improve clinical outcomes in patients at risk of post cardiac surgery AKI.

## Introduction

Acute kidney injury (AKI), defined clinically by an acute reduction in glomerular filtration rate (GFR) and rises in serum creatinine [1-3], complicates 25 to 30% of cardiac surgery procedures, is associated with an in-hospital mortality of 10%, and increases in-hospital resource use of up to 100% [2-4]. Our understanding of the underlying processes is poor and the prognosis for patients with AKI has remained essentially unchanged for decades.

Recently, a number of epidemiological studies have reported improved outcomes in critically ill patients who are obese when compared with normal weight individuals [5]. This phenomenon, termed the obesity paradox, includes improved outcomes in intensive care patients with acute kidney injury [6], as well as following cardiac surgery [7,8]. A beneficial effect of obesity on inflammatory organ injury is not universally accepted, however; other authors have shown that very obese patients are at increased risk of AKI following cardiac surgery [9]. There is also no clear mechanistic explanation, and the findings of these studies have been variously attributed to statistical bias [10], confounding and reverse epidemiology [11], the anti-inflammatory effects of adipose tissue [12,13], and the thrifty genotype [14].

We have described a novel large animal recovery model of post-cardiopulmonary bypass (post-CPB) AKI in swine [15] that shows quantitative and qualitative homology to the renal injury and dysfunction observed in cardiac surgery patients [16]. Renal inflammation, endothelial dysfunction and refractory cellular hypoxia are central features of AKI in this model [17,18]. In a separate study we noted significant adiposity in domestic swine fed an atherogenic high-fat diet [19]. To investigate the mechanisms underlying the obesity paradox we examined the effects of obesity caused by high-fat feeding on post-CPB AKI in the swine recovery model using an index of GFR as our primary outcome and measures of endothelial homeostasis and inflammation as secondary outcomes.

## Materials and methods

Twenty-eight female Large White Landrace crossbred pigs approximately 4 months old and weighing 50 to 70 kg were used. Animals received care in accordance with and under license of the Animals (Scientific Procedures) Act 1986 and conforming with the Guide for the Care and Use of Laboratory Animals published by the US National Institutes of Health (NIH Publication No. 85–23, revised 1996). The study had received local (University of Bristol) institutional review board approval, and was conducted under UK Home Office License PPL 30/2522.

### Intervention

We allocated animals to four groups. Group 1 was sham operation (surgical dissection with 2.5 hours of general anaesthesia) in swine fed a 12-week normal chow diet (*n* = 8; Sham). Group 2 was CPB (2.5 hours) in swine fed a 12-week normal chow diet (*n* = 8; CPB). Group 3 was sham operation in swine fed a 12-week high-fat diet containing 15% lard, 1.5% cholesterol and 1% cholic acid using a protocol we have previously shown to be atherogenic [19] (*n* = 6; Sham + HF). Group 4 was CPB (2.5 hours) in swine fed a 12-week high-fat diet for 12 weeks (*n* = 6; CPB + HF).

Anaesthesia and CPB were performed as described previously [17,18]. Serum cholesterol levels were determined by measurement of plasma total cholesterol from blood samples at 2, 4 and 12 weeks after commencement of diet with an automatic analyser (Eastman Kodak Co., Rochester, NY, USA). After 12 weeks of high-fat or normal chow diet, pigs were weighed and anaesthetised with: induction anaesthesia, ketamine (10 mg/kg, ketaset) and halothane (2.0 to 4.0%); and maintenance anaesthesia, halothane (1.0 to 2.0%) with nitrous oxide 50% in oxygen. Depth of anaesthesia was assessed by respiratory and pulse rates, ocular reflexes including the palpebral response and ocular position. No muscle relaxant was administered. Animals were intubated and positive-pressure ventilation was commenced in a circle circuit using a Penlon Nuffield 200 (Abingdon, Oxford, UK) initially to achieve peak inspiratory pressures of 30 cmH_2_O in a 1:4 (inspiratory:expiratory) ratio with modifications to maintain partial pressure of carbon dioxide between target values of 35 and 45 mmHg. Lung compliance, partial pressure of oxygen (PaO_2_)/fraction of inspired oxygen (FiO_2_) ratio, and work of breathing were measured *in vivo* at baseline, 1.5 hours and 24 hours post intervention using the SERVO-i Universal Ventilator (Maquet Gmbh, Rastatt, Germany) and volume-controlled ventilation with a tidal volume of 10 ml/kg, FiO_2_ of 0.5, respiratory rate of 12 breaths/minute and peak end-expiratory pressure of 5 cm/H_2_O.

Intravenous heparin (20,000 IU) and cefuroxime (750 mg) were administered at the start of the experimental period. The pre-intervention and post-intervention period central venous pressure (8 to 12 mmHg), hydration, and sodium load (500 ml/hour, 0.9% normal saline) were strictly controlled. Venous access and measurement of central venous pressure was achieved through direct puncture of the left external jugular vein using a quad-lumen central venous catheter (MultiCath4 Expert; Vygon GmbH, Aachen, Germany). Arterial blood pressure was continuously monitored via a 20G Vygon catheter placed in the left common carotid artery. Core body temperature was assessed using a rectal temperature probe. Intramuscular buprenorphine (vetergesic) was administered for pain relief. Cardiopulmonary bypass was performed using a minimally invasive approach. This avoided the need for a median sternotomy and was chosen to allow safe animal recovery. CPB venous drainage was established via a 24Fr Smart Cannula® (Smartcanula LLC, Lausanne, Switzerland) placed in the right external jugular vein and advanced to the right atrium. Arterial return was achieved via a 14Fr Smart Cannula® placed in the right internal carotid artery and advanced to the brachiocephalic trunk. All animals received heparin 300 IU/kg. The CPB circuit was primed with Hartman’s solution (2,000 ml) and heparin (5,000 IU). Normothermic (38 to 39°C in pigs), nonpulsatile CPB was commenced by a qualified clinical perfusionist using a Stöckert Multiflow Roller Pump (Sorin Group GmbH, Munich, Germany) to achieve a target flow rate of 80 to 90 ml/kg/minute of blood through the hollow fibre–membrane oxygenator apparatus (Dideco D708 Compact-Flo; Sorin Biomedica, Via Crescentio, Italy). Mean arterial blood pressure was maintained between 65 and 75 mmHg with small incremental doses of the alpha-adrenergic agonist metaraminol, FiO_2_ at 50% and partial pressure of carbon dioxide between 35 and 45 mmHg. Total CPB (or sham intervention) time was 2.5 hours. This has been shown to result in significant kidney injury in our previous work [17,18] and is representative of a prolonged CPB duration, a recognised risk factor for AKI in clinical studies [3]. Post-bypass animals are recovered, then re-anaesthetised and outcomes re-evaluated after 24 hours with nephrectomy for tissue analyses performed prior to euthanasia.

### Biochemical markers of renal injury

Creatinine clearance was calculated from urinary and serum creatinine values and urinary volumes collected over 90 minutes, at three time points: pre-intervention, immediately post intervention and at 24 hours post intervention, as described previously [17,18]. Serum and urine creatinine values were determined with a commercial reagent kit (HiCo Creatinine; Boehringer Mannheim GmbH Diagnostica, Lewes, UK). Levels of inflammation were determined by measurement of endothelin-1 (ET-1) levels in renal tissue as described below, and the measurement of urinary neutrophil gelatinase-associated lipocalcin (NGAL; Bioporto Diagnostics A/S, Gentofte, Denmark) and serum interleukin (IL)-6 (Invitrogen, Paisley, UK) using enzyme-linked immunosorbent assay.

### Endothelial function

To assess renal endothelial function at 24 hours, we measured *in-vivo* renal artery blood flow, and the response to suprarenal intra-aortic acetylcholine (endothelial-dependent response) or sodium nitroprusside (endothelial-independent response) infusion. Renal blood flow was measured directly by placing a transonic perivascular flowprobe (Oxford Optronix, Oxford, UK) around the left renal artery, which was accessed via a mini-laparotomy. The flowprobe was connected to a T106 Transonic flow meter (Transonics Systems, Ithaca, NY, USA) with in-built calibrations, which was in turn connected to a laptop containing powerlab software (ADInstruments, Dunedin, NZ) that gave an output of renal blood flow in litres per minute. To avoid vasoconstriction (as a result of manipulation) confounding our results, a 10-minute rest or nonmanipulation period was provided prior to baseline measurements. This was followed by a 5-minute period of baseline flow measurements and then infusion of acetylcholine or sodium nitroprusside for 5 minutes during which flow was continuously monitored. The mean flow during the time period was calculated and flow measurements were performed in triplicate both with and without acetylcholine/sodium nitroprusside. Endothelial dysfunction was determined by the change in renal blood flow in response to an acetylcholine infusion (0.1 to 10 μg/kg/minute) administered via a 14G peripheral venous cannula (BD Venflon, Becton Dickinson, Oxford, UK) inserted into the suprarenal abdominal aorta. Sodium nitroprusside (0.1 to 10 nmol/kg/minute) was used to control for endothelium-independent relaxation. Renal cortical and medullary nucleotide levels were measured using reverse-phase high-performance liquid chromatography as described previously [17,18].

### Renal tissue

Using formalin-fixed, paraffin-embedded, or snap-frozen 5 μm transverse renal sections, immunocytochemistry using the Vector avidin–biotin complex method (Vector Laboratories, Peterborough, UK) or immunofluorescence was performed for ET-1 (Acris, Herford, Germany), a marker of inflammation, and endothelial nitric oxide synthase (Santa Cruz Biotechnology, Heidelberg, Germany), a marker of endothelial homeostasis, inflammatory cells, (MAC-387; Abcam, Cambridge, UK), and cell proliferation (proliferating cell nuclear antigen; Sigma, St Louis, MO, USA). Cell staining density was determined by counting the number of cells in a 0.125 mm^2^ area. Vascular endothelium was identified by percent staining of immunocytochemistry for biotinylated dolichos biflorus agglutinin lectin (Vector Laboratories). Levels of apoptosis were determined by western blotting for cleaved caspase-3 (Calbiochem, UK), as were levels of expression of markers of renal inflammation, and cell survival ET-1 (Acris), p65 nuclear factor-kappa B (NF-κB; Cell Signaling Technology, Beverly, MA, USA), inducible nitric oxide synthase (iNOS; Thermo Fisher Scientific, Waltham, MA, USA), vascular endothelial growth factor (VEGF; Calbiochem), and B-cell lymphoma-2 (Bcl2; Cell Signaling Technology), Akt, phospho-Akt (Ser473) and p70S6K (all Cell Signaling Technology), and hypoxia inducible factor (HIF)-1 alpha (Sigma), as described previously [17,18].

### Power of the study and statistical analysis

Creatinine clearance, which we have previously validated as an index of GFR in this model (correlation coefficient 0.74 to 0.78) [15,17], was the primary endpoint. We calculated that a study with 24 animals (six per group) would have a 90% power to detect a large effect size of 0.7 standard deviations, equivalent to a difference of 16.5 ml/minute in creatinine clearance between groups assuming a within-group standard deviation of 23.5. Extra animal experiments (*n* = 4) were added to some groups to complete endothelial function testing in at least four animals per group. Data are expressed throughout as mean (standard error of the mean) or median (interquartile ranges) and treatment differences are reported as mean difference (95% confidence interval). Differences between groups were calculated using analysis of variance with adjustment for baseline differences for repeated measures. Non-normally distributed data were log transformed with effect estimates reverse transformed to express effect size and CIs on a linear scale. *P* <0.05 was considered statistically significant. All analyses were carried out using SPSS 14.0 (SPSS Inc., Chicago, IL, USA).

## Results

**Figure 1:**
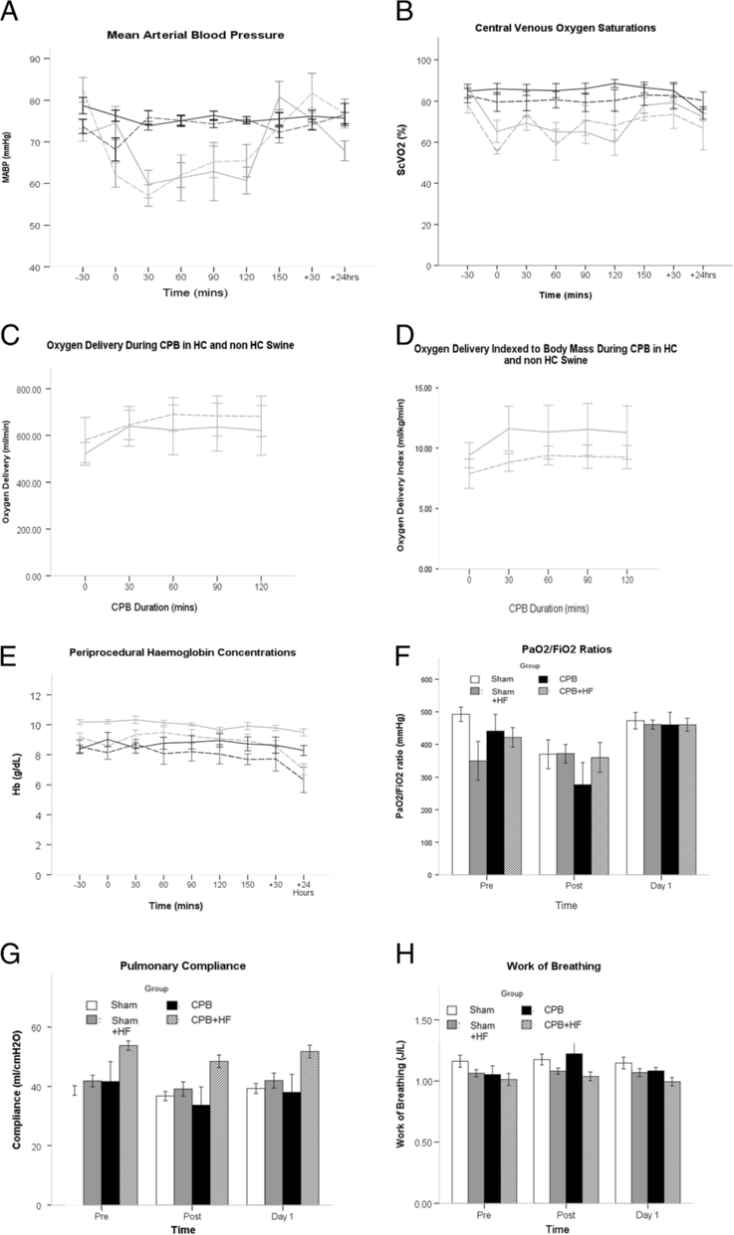
**Anaesthesia and cardiopulmonary bypass. (A)** Mean arterial blood pressure (MABP). **(B)** Central venous oxygen saturation (ScVO_2_). **(C)** Global oxygen delivery (DO_2_) during cardiopulmonary bypass (CPB). **(D)** DO_2_ during CPB adjusted for body mass. **(E)** Periprocedural haemoglobin (Hb) concentrations. **(F)** Periprocedural partial pressure of oxygen/fraction of inspired oxygen (PaO_2_/FiO_2_) ratios. **(G)** Lung compliance. **(H)** Work of breathing. Key for line diagrams: black solid, Sham; black hatched, Sham + HF; grey solid, CPB; grey hatched, CPB + HF. Values represent mean (standard error of the mean), at least *n* = 6 per group. For graphs, pooled estimates for pairwise comparisons derived from analysis of variance for repeated measures and adjusted for mean baseline. Estimates for baseline values were as follows: MABP, 79 mmHg; ScVO_2_, 83.4%; DO_2_ 556 ml/minute; Hb, 9.1 g/dl; PaO_2_/FiO_2_ 386 mmHg, compliance 43.2 J/l. MABP: Sham–Sham + HF, 1.5 mmHg (95% CI = −5.5 to 8.5), *P* = 1.0; Sham–CPB, 12.6 mmHg (95% CI = 6.8 to 18.5), *P* <0.001; Sham–CPB + HF, 7.9 mmHg (95% CI = 0.3 to 15.4), *P* = 0.037; CPB–CPB + HF, −4.7 mmHg (95% CI = −3.3 to 12.8), *P* = 0.654; test for overall treatment effect for MABP, *P* <0.001; test for Time* Group interaction, *P* <0.001. ScVO_2_: Sham–Sham + HF, 3.3% (95% CI = −4.8 to 11.4), *P* = 1.0; Sham–CPB, 20.4% (95% CI = 11.7 to 29.0), *P* <0.001; Sham–CPB + HF, 15.2% (95% CI = 5.3 to 25.2), *P* = 0.002; CPB–CPB + HF, −5.1% (95% CI = −16.5 to 6.2), *P* = 1.0; test for overall treatment effect for ScVO_2_, *P* <0.001; test for Time* Group interaction, *P* = 0.134. Oxygen delivery: CPB–CPB + HF, 0.69 ml/minute (95% CI = −199 to 201), *P* = 0.994; test for Time* Group interaction, *P* = 0.184. Oxygen delivery index: CPB–CPB + HF, 0.95 ml/kg/minute (95% CI, −4.4 to 2.5), *P* = 0.534; test for Time* Group interaction, *P* = 0.505. Hb: test for overall treatment effect, *P* = 0.351; test for Time* Group interaction, *P* = 0.653. PaO_2_/FiO_2_: test for overall treatment effect, *P* = 0.393; test for Time* Group interaction, *P* = 0.486. Compliance: test for overall treatment effect, *P* = 0.381; test for Time* Group interaction, *P* = 0.098. Work of breathing: test for overall treatment effect, *P* = 0.295; test for Time* Group interaction, *P* = 0.086. CI, confidence interval; HF, high-fat diet.

Anaesthesia, recovery and reassessment were completed in all animals. Baseline characteristics are presented in Table 1. Pigs fed a high-fat diet were significantly heavier compared with those fed a normal chow diet. Weight gain was attributed in significant part to the accumulation of adipose tissue on the trunk (>2 cm), neck (>3 cm) and surrounding the viscera, which was not evident in pigs fed a normal chow diet. A high-fat diet also resulted in significant hyperlipidaemia (Table 1). There was no difference between the groups for baseline creatinine clearance. During the 2.5-hour intervention time period, CPB resulted in lower perfusion pressures and lower central venous oxygen saturation values compared with Sham pigs, with no difference between CPB and CPB + HF groups, or between Sham and Sham + HF groups for these measures (Figure 1A,B). There was no significant difference between CPB and CPB + HF pigs in terms of global oxygen delivery (Figure 1C) or oxygen delivery indexed to body mass during CPB (Figure 1D). Pigs undergoing bypass required significantly higher doses of vasopressor compared with pigs undergoing Sham procedures (Table 1); however, doses administered to CPB and CPB + HF groups were not significantly different. There were no differences between the groups with respect to periprocedural haemoglobin concentrations or gas exchange (PaO_2_/FiO_2_ ratio) (Figure 1E,F). Lung compliance was higher in the CPB + HF group at baseline, but no treatment effect on compliance was detected between the groups post intervention or for work of breathing (Figure 1G,H).

**Table 1:** Baseline data

**Variable**	**Sham**	**Sham + HF**	**CPB**	**CPB + HF**	**ANOVA**
	**(*n*** **= 8)**	**(*n*** **= 6)**	**(*n*** **= 8)**	**(*n*** **= 6)**	***P *** **value**
Weight (kg)	57.0 (1.4)	66.2 (4.1)	56.4 (1.4)	72.9 (1.8)	0.001
Serum creatinine (μmol/l)	138.8 (9.7)	136.2 (5.9)	132.3 (7.0)	136.8 (5.6)	0.937
Urine output (ml/kg/hour)	1.1 (0.17)	1.3 (0.22)	1.9 (0.40)	1.6 (0.30)	0.209
Creatinine clearance (ml/minute)	103.2 (8.2)	116.5 (17.1)	116.4 (12.9)	122.7 (19.9)	0.791
Creatinine clearance adjusted for body weight (ml/minute/kg)	1.7 (0.2)	1.6 (0.2)	2.1 (0.2)	1.6 (0.3)	0.327
Serum total cholesterol (mmol/l)	1.5 (0.9)	15.3 (2.5)	1.5 (0.8)	12.8 (3.4)	<0.001
Mean arterial blood pressure (mmHg)	81.1 (2.6)	73.7 (1.7)	82.4 (2.4)	71.7 (1.5)	0.062
Perioperative fluid administration (ml)	5,000 (4,750 to 5,000)	5,000 (5,000 to 5,000)	5,500 (5,000 to 6,000)	5,125 (3,500 to 5,500)	0.230
Baseline haemoglobin (g/dl)	8.7 (0.4)	10.1 (0.2)	8.9 (0.3)	9.2 (0.6)	0.064
Perioperative metaraminol dosage (mg)	0 (0 to 0)	0 (0 to 0)	5.5 (0.5 to 15)	13.0 (10.0 to 17.0)	0.007
Baseline lung compliance (mmHg*l)	38.7 (1.6)	41.9 (2.0)	41.7 (6.7)	53.8 (1.6)	0.01
Baseline PaO_2_/FiO_2_ (mmHg)	464 (440 to 540)	412 (376 to 416)	454 (400 to 488)	450 (374 to 476)	0.355
Work of breathing (J/l)	1.2 (0.05)	1.1 (0.03)	1.1 (0.07)	1.0 (0.05)	0.148

Data presented as mean (standard error of the mean) or median (interquartile range). Pairwise comparisons derived from analysis of variance (ANOVA) for weight were as follows: Sham–Sham + HF, −9.2 kg (95% confidence interval (CI) = −17.2 to −1.2), *P* = 0.016; Sham–CPB, 0.6 kg 95% CI = −5.8 to 6.9), *P* = 1.0; Sham–CPB + HF, −15.9 kg (95% CI = −24.3 to −7.4), *P* <0.001; CPB–CPB + HF, −16.7 kg (95% CI = −25.1 to −7.7), *P* <0.001. Pairwise comparisons derived from ANOVA for metaraminol dose were as follows: Sham–Sham + HF, 0.27 mg (95% CI = −10.4 to 10.9), *P* = 0.959; Sham–CPB, −11.9 mg (95% CI = −20.9 to −2.9), *P* = 0.012; Sham–CPB + HF, −14.1 (95% CI = −23.9 to −4.3), *P* = 0.006; CPB–CPB + HF, 2.3 mg (95% CI = −12.3 to 7.7), *P* = 0.651. CPB, cardiopulmonary bypass; HF, high-fat diet; PaO_2_/FiO_2_, partial pressure of oxygen/fraction of inspired oxygen.

### Sham operated pigs fed a high-fat diet

**Figure 2:**
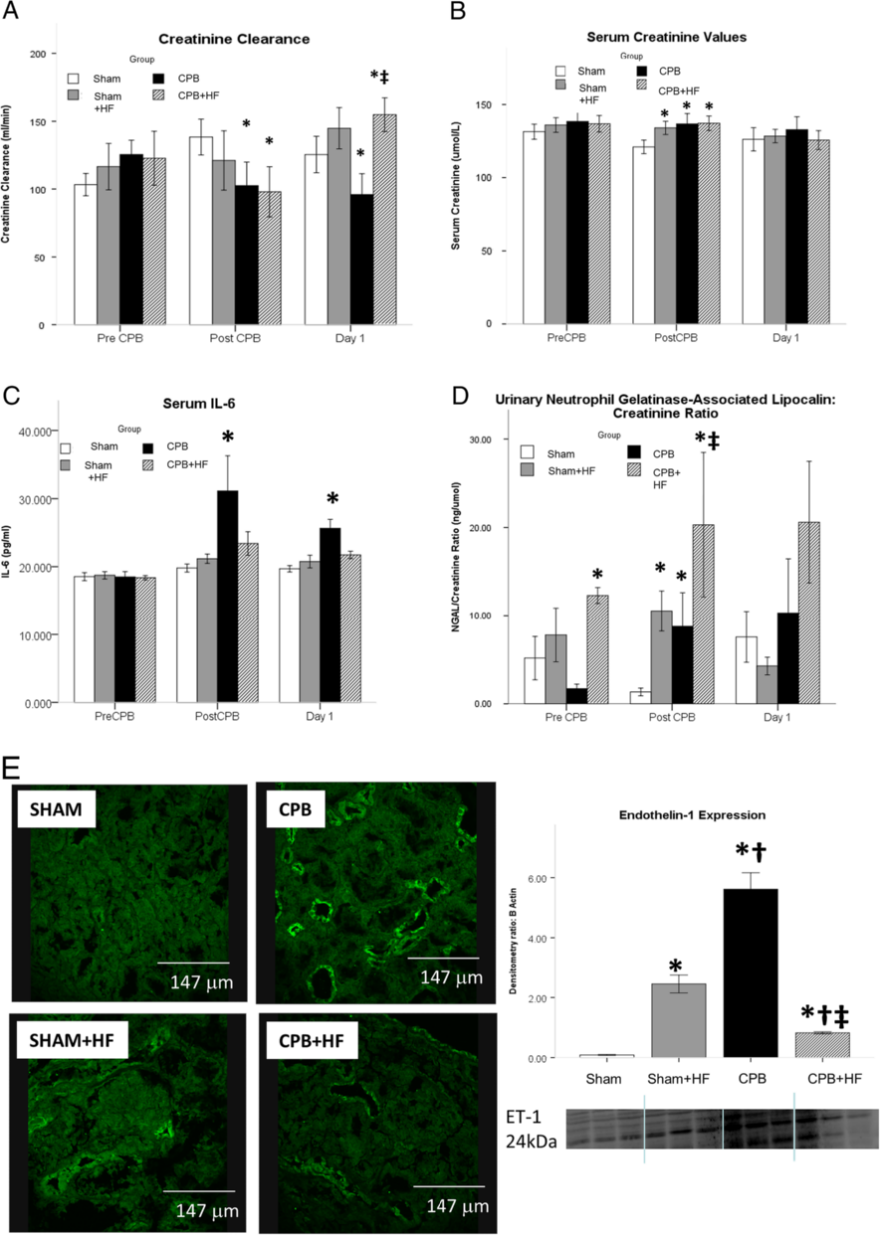
**Biochemical Markers of AKI. (A)** Creatinine clearance, an index of GFR, as per Figure 1A. **(B)** IL-6, a serum biomarker of AKI, **(C)** Serum Creatinine, **(D)** IUrinary Neutrophil Gelatinase-Associated Lipocalcin (NGAL), a urinary AKI biomarker . Values represent mean (SEM) at least *n *= 6 per group. **(E)** Immunofluorescence for tubular Endothelin-1 expression at 24 Hours post Intervention, quantified by Western Blotting and densitometry as described. Values represent mean (SEM) at least *n* = 6 per group. *p <0.05 versus sham, †*p* <0.05 versus Sham+HF, ‡*p* <0.05 versus CPB. For graphs pooled estimates for pairwise comparisons derived from Analysis of Covariance with adjustment for baseline Creatinine Clearance estimated at 114 ml/min, serum creatinine 135 µmol/L, serum IL-6 estimated at 18.6 pg/ml, urinary NGAL creatinine ratio estimated at 3.69 ng/µmol, were as follows: **Creatinine Clearance;** Post CPB Intervention: Sham- Sham + HF, 25.9 ml/min (95% CI -19.4 to 71.1), *p* = 0.249, Sham-CPB 50.2 ml/min (95% CI 5.9 to 94.4), *p* = 0.028, Sham-CPB + HF 52.9, (95% CI 7.1 to 98.6), *p* = 0.026. Test for overall treatment effect *p* = 0.079. Day 1 Post intervention: Sham-Sham+HF, -14.8 ml/min (95% CI 56.7 to 27.1), *p *= 472, Sham-CPB 42.7 ml/min (95% CI 3.8 to 81.5), *p *= 0.033, Sham-CPB + HF -22.6 ml/min, (95% CI -65.0 to 19.7), *p* = 0.280, CPB-CPB + HF -65.3 ml/min (95% CI -106.9 to -23.7) *p* = 0.004. Test for overall treatment effect *p* <0.001. **Serum Creatinine;** Post CPB Intervention: Sham-Sham + HF, -9.5 µmol/L (95% CI -17.2 to -1.7), *p* = 0.019, Sham-CPB -9.9 µmol/L (95% CI -17.5 to -2.4), *p* = 0.012, Sham-CPB + HF -11.9 µmol/L, (95% CI -20.1 to -3.9), *p* = 0.006. Test for overall treatment effect *p* = 0.017. Day 1 Post intervention: Test for overall treatment effect *p* = 0.788. **Serum IL-6;** Post Intervention: Sham-Sham + HF -1.5 pg/ml (95% CI -9.3 to 6.4), *p* = 0.701, Sham-CPB -11.3 (95% CI -19.6 to -3.2), *p* = 0.009, Sham-CPB + HF -3.5, (95% CI -12.1 to 5.0), *p* = 0.399. Test for overall treatment effect *p* = 0.039. Day 1 Post Intervention: Sham-Sham + HF, -1.0 pg/ml (95% CI -3.7 to 1.7), *p* = 0.433, Sham-CPB -5.9 pg/ml (95% CI -8.7 to -3.2), *p* <0.001, Sham-CPB + HF -2.1 pg/ml, (95% CI -4.9 to 0.83), *p* = 0.153. Test for overall treatment effect *p* = 0.001. **Urinary NGAL/ Creatinine Ratio;** Post Intervention: Sham-Sham + HF, 0.11 (95% CI 0.04 to 0.37), *p* = 0.001, Sham-CPB 0.16 (95% CI 0.04 to 0.66), *p* = 0.014, Sham-CPB + HF 0.09, (95% CI 0.02 to 0.40). Test for overall treatment effect *p* = 0.004. Day 1 Post Intervention: Test for overall treatment effect *p* = 0.601.

**Figure 3:**
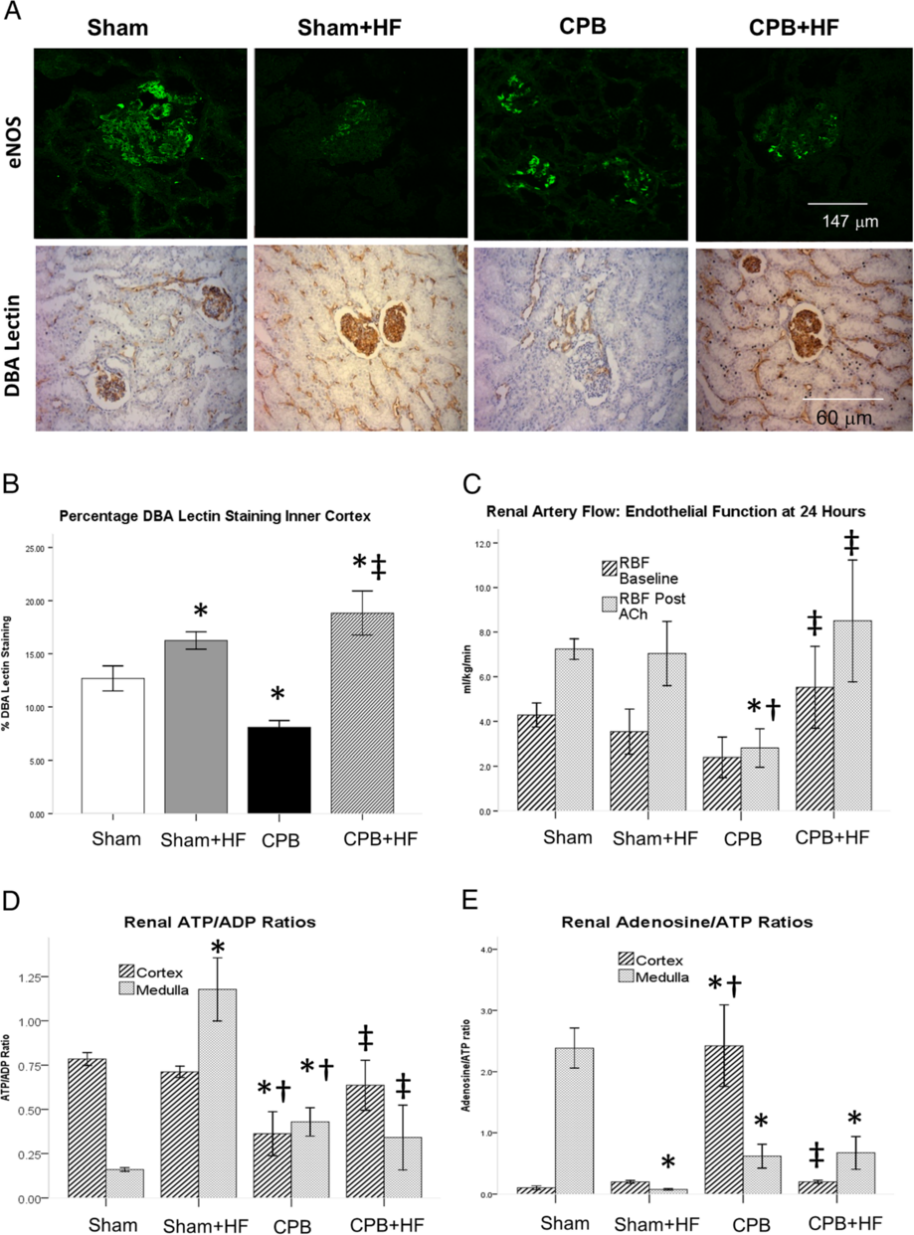
**Endothelial homeostasis (A) Immunofluorescence for glomerular endothelial nitric oxide synthase (eNOS) at 24 hours post intervention and immunocytochemistry (ICC) for dolichos biflorus agglutinin (DBA) lectin.** eNOS staining was almost completely absent in high-fat diet (HF) swine. Glomerular eNOS staining in cardiopulmonary bypass (CPB) swine was reduced and this was associated with endothelial dysfunction and reduced glomerular filtration rate (GFR). DBA lectin staining was reduced in CPB pigs indicative of glycocalyceal loss but was preserved in CPB + HF pigs indicating maintenance of endothelial homeostasis. There was increased microvascular staining density in HF pigs that may underlie the preservation of renal blood flow observed in CPB + HF pigs. **(B)** Quantification of ICC for DBA lectin staining expressed as percentage staining at 24 hours post injury. **(C)** Renal blood flow/kilogram body weight (RBF) measured on a single renal artery via laparotomy. NO dependent vascular reactivity was determined by the change in renal blood flow in response to an infusion of acetylcholine (RBF post Ach). **(D)** ATP and **(E)** adenosine levels, expressed as ratios in the renal cortex and medulla at 24 hours post intervention. Values represent mean (standard error of the mean) of at least *n* = 4 per group. *P <0.05 versus sham, †*P* <0.05 versus Sham + HF, ‡*P* <0.05 versus CPB.

**Figure 4:**
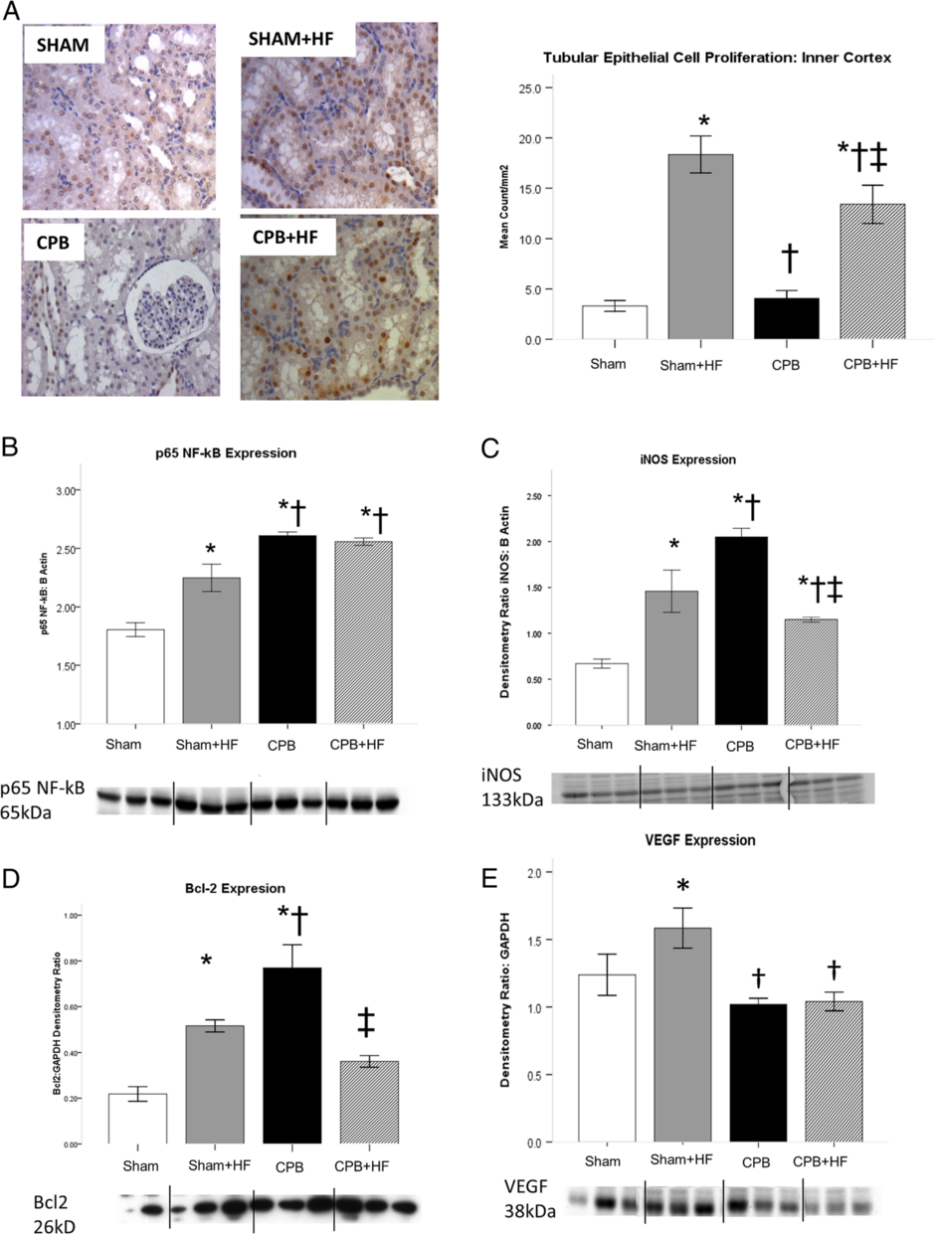
**Proliferation and p60 NF-κB signalling. (A)** Proliferating tubular epithelial cell (proliferating cell nuclear antigen-positive) count in the inner cortex at 24 hours post intervention. **(B)** p60 nuclear factor-kappa B (NF-κB) expression, and expression of proteins encoded by the NF-κB-dependent genes: **(C)** Inducible nitric oxide synthase (iNOS). **(D)** B-cell lymphoma protein 2 (Bcl2). **(E)** Vascular endothelial growth factor (VEGF). VEGF expression was increased in Sham + HF pigs, was also associated with increased microvascular density. Values derived from densitometry of western blots and expressed as a ratio to β-actin or glyceraldehyde-3-phosphate dehydrogenase (GAPDH). Representative blots are shown. Values represent mean (standard error of the mean) of at least *n* = 4 per group. *P <0.05 versus sham, †*P* <0.05 versus Sham + HF, ‡*P* <0.05 versus cardiopulmonary bypass (CPB). HF, high-fat diet.

**Figure 5:**
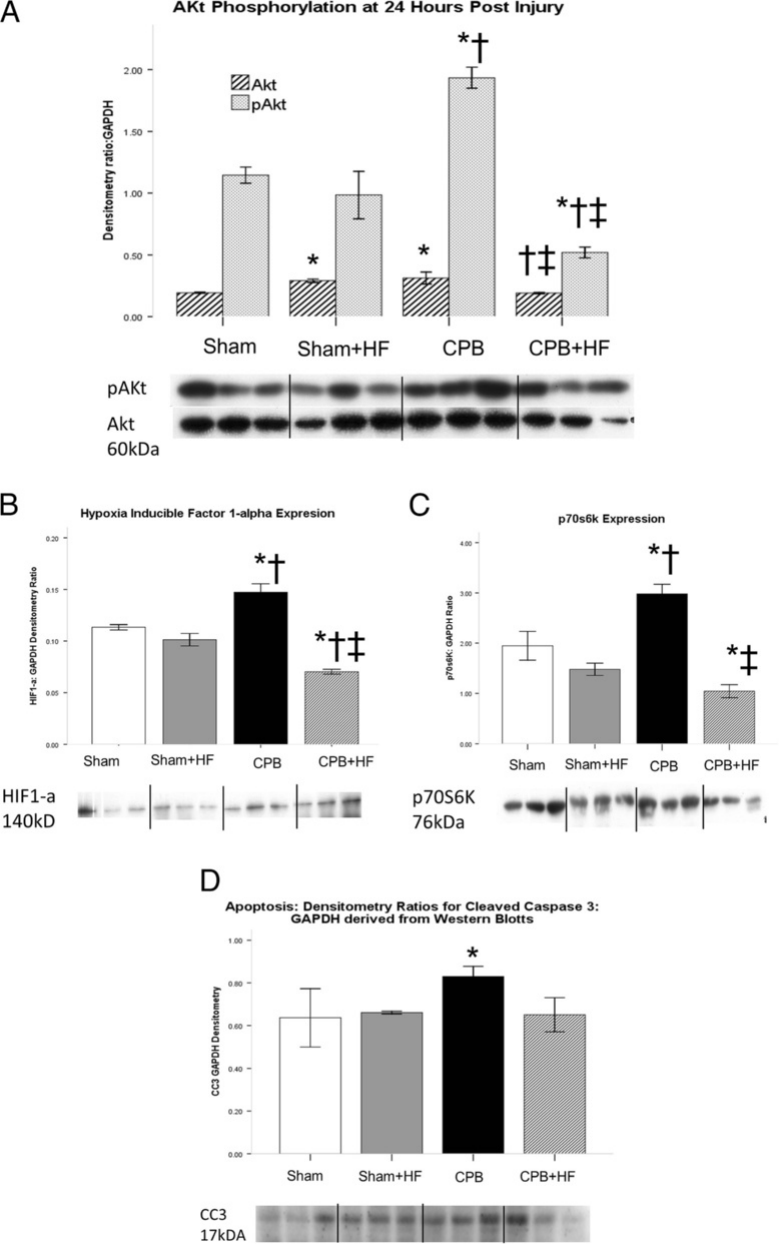
**Survival signalling and apoptosis. (A)** Pro-survival signalling; expressed as pAKt:Akt ratio and levels of downstream effectors. **(B)** Hypoxia inducible factor (HIF)-1 alpha. **(C)** p70S6K, a signalling protein in the Akt/mTOR pathway. **(D)** Apoptotic signalling as indicated by cleaved caspase 3 (CC3) levels, with values derived from densitometry of western blots and expressed as a ratio to β actin or glyceraldehyde-3-phosphate dehydrogenase (GAPDH). Representative blots are shown. Values represent mean (standard error of the mean) of at least *n* = 4 per group. *P <0.05 versus sham, †*P* <0.05 versus Sham + HF, ‡*P* <0.05 versus CPB.

Sham + HF pigs demonstrated similar creatinine clearance over the 24-hour follow-up period compared with non-HF Sham pigs (Figure 2A). Serum creatinine levels were also similar at 24 hours post intervention (Figure 2B). Measures of inflammation in serum IL-6 were not increased by a high-fat diet (Figure 2C) although specific markers of renal inflammation, urine NGAL levels and intrarenal ET-1 expression, were significantly elevated post intervention (Figure 2D,E). NGAL levels were significantly elevated immediately post intervention, but intergroup differences were no longer significant at 24 hours. Tissue analysis at 24 hours showed ET-1 co-localised to pseudo-dilated renal tubules. We have shown previously that this is indicative of epithelial cell stress [17]. The high-fat diet resulted in inflammation that was associated with marked reductions in endothelial nitric oxide synthase expression and significant increases in microvascular staining (Dolichos biflorus agglutinin lectin) density (Figure 3A,B). Renal artery flows and endothelial responsiveness to acetylcholine were unchanged from non-HF Sham pigs at 24 hours although there were differences in regional purine nucleotide levels; medullary ATP levels were increased and levels of adenosine were reduced (Figure 3C,D,E). We have shown previously that these changes are indicative of alterations in regional perfusion [18,19]. In high-fat diet pigs, inflammation was associated with high rates of tubular epithelial proliferation (Figure 4A) and increased p65 NF-κB expression (Figure 4B), as well as increased expression of the proteins encoded by several NF-κB-dependent genes: iNOS, VEGF and Bcl2 (Figure 4C,D,E). The high-fat diet did not alter pro-survival pAkt/p70S6K/HIF-1α signalling (Figure 5A,B,C) or levels of cleaved caspase 3, a marker of apoptosis relative to non-HF Sham pigs (Figure 5D).

### Cardiopulmonary bypass in pigs fed a normal diet

CPB in pigs fed a normal diet resulted in AKI characterised by sustained reductions in creatinine clearance and increases in the serum biomarker IL-6 over the 24-hour follow-up period (Figure 2A,C). These changes were not reflected in altered serum creatinine values at 24 hours (Figure 2B). CPB also resulted in significant increases in markers of renal inflammation, urinary NGAL at 1.5 hours and intrarenal ET-1 at 24 hours, compared with Sham operated non-HF controls (Figure 2D,E). Levels of ET-1 expression and serum IL-6 were significantly greater in these pigs relative to all other experimental groups, but urine NGAL levels were found to be lower than those in the high-fat groups. CPB resulted in significant loss of endothelial nitric oxide synthase and vascular endothelial staining density (Figure 3A,B). CPB also resulted in endothelial dysfunction, as indicated by an attenuation of renal endothelial responsiveness to acetylcholine, depletion of ATP and increased adenosine in the renal cortex and reciprocal increased ATP and reduced adenosine in the renal medulla (Figure 3C,D,E). CPB was associated with increased p65 NF-κB and iNOS expression and increased tubular proliferation rates (Figure 4A,D,C). Unlike Sham + HF pigs, VEGF was not increased (Figure 4E), and this is perhaps attributable to the more severe inflammation evident in this group. Levels of the apoptosis marker cleaved caspase 3 were increased relative to other groups despite a significant increase in pro-survival pAkt/p70S6K/HIF-1α/Bcl 2 signalling (Figure 5A,B,C,D).

### Cardiopulmonary bypass in pigs fed a high-fat diet

In CPB + HF swine, creatinine clearance was reduced immediately post CPB; however, unlike in CPB-only pigs, this was not associated with increases in serum IL-6 (Figure 2A,C) and by 24 hours creatinine clearance had recovered and was similar to those in both Sham groups. ET-1 levels were reduced relative to that observed in CPB-only pigs (Figure 2E). Renal blood flow was increased at 24 hours, in association with preservation of microvascular density relative to Sham + HF pigs, and endothelial homeostasis was preserved as determined by renal blood flows and endothelial responsiveness to acetylcholine (Figure 3). p65 NF-κB expression was increased relative to Sham-only pigs, as was expression of iNOS, and the rate of tubular epithelial cell proliferation (Figure 4A,B,C). VEGF and Bcl2 expression were reduced (Figure 4D,E), however, as was pro-survival pAKt/p70S6K/HIF-1α signalling (Figure 5A,B,C). Levels of the apoptosis marker cleaved caspase 3 were similar to those in Sham groups (Figure 5D).

## Discussion

A 12-week high-fat diet resulted in significant obesity and hyperlipidaemia in swine. This promoted inflammatory cell signalling, vascular endothelial homeostasis, and epithelial cell survival in swine kidneys, preserved renal blood flow and GFR, and prevented CPB-mediated injury.

This study has several strengths, in particular the ability to evaluate functional, biochemical and histological changes in kidneys in response to clinical stimuli such as CPB, or obesity, in a large animal recovery model of AKI where the physiology has significant homology to that observed in humans [15-18]. An important finding from this study is the clear association between acutely elevated serum IL-6 levels, a candidate biomarker for AKI currently being evaluated in clinical studies [20], and persistent reductions in GFR at 24 hours that are associated with ongoing inflammation and hypoxia. In contrast to IL-6, urinary NGAL was increased by a HF diet and did not discriminate between pigs that did or did not develop sustained reductions in GFR. This is consistent with false positives of this AKI biomarker in clinical studies in patients with pre-existing inflammatory states such as chronic kidney disease [21]. The acute (within 1.5 hours) elevation in IL-6 also highlights the limitations of existing clinical definitions of AKI that are based on peak serum creatinine levels detected up to 48 hours post injury [1,2]. Our model does not show significant rises in serum creatinine at 24 hours post CPB, indicating a modest degree of injury. This contrasts with models of AKI in swine that involve periods of arterial occlusion and reperfusion (ischaemia and reperfusion), which can demonstrate acute rises in serum creatinine within hours [22,23]. This picture is not typical of post-cardiac surgery AKI, however; clinical studies indicate that serum creatinine does not rise for up to 48 hours post surgery in patients with AKI [24]. Warm ischaemia and reperfusion for periods of up to 2 hours elicits a severe renal injury, with measured mean creatinine clearance of 7 ml/minute 4 hours following reperfusion in one study [22]. This is not consistent with measured creatinine clearances that we have documented after cardiac surgery [16]. Together, these studies underpin the value of translational models that can reflect different clinical scenarios. In the case of post-CPB AKI, our work indicates an inflammatory pathogenesis as opposed to ischaemia and reperfusion caused by periods of arterial occlusion where tubular necrosis is more marked [22,23].

Epidemiological studies have speculated as to the causal pathway between obesity and improved clinical outcomes in the critically ill, with some reports suggesting that these observations may be attributed to attenuation of the inflammatory response [12,13]. In contrast, our results indicate that renal protection was conferred by increased renal inflammation attributable to an obesogenic high-fat diet. This apparent paradox merits a careful consideration of the limitations of the current study. AKI in young healthy female swine may not reflect the multifactorial AKI observed in older cardiac surgery patients. For example, atherosclerosis, a common finding in the older cardiac patient, has been shown to alter inflammation and repair mechanisms in swine models of renal ischaemia and reperfusion [22,23]. Weight gain in young swine may also be attributed to increase in muscle as well as adipose tissue, although adiposity was marked in these animals. Serum lipid levels were higher than those commonly recorded in humans. The principal limitation of this study is that we cannot differentiate the effects of obesity as distinct from the effects of hyperlipidaemia on outcomes. Previous work has demonstrated that hypercholesterolaemic homozygous apolipoprotein-E-deficient mice fed a high-fat diet are resistant to ischaemic AKI [25] in the absence of obesity. Similarly, pigs fed a high-fat diet that develop hyperlipidaemia in the absence of significant weight gain are protected against myocardial ischaemia reperfusion injury [26]. In that study, similar to our own findings, the protective effects of high-fat feeding and hyperlipidaemia in porcine myocardium were attributed to preserved endothelial homeostasis and increased microvascular density in the presence of inflammation and oxidative stress [26]. This suggests that it is the chronic renal inflammation consequent to hyperlipidaemia which protects against post-CPB AKI. Obesity may in fact represent an epiphenomenon; a bystander also attributable to high-fat feeding that is more readily measureable in epidemiological analyses, and this is reflected by the assessment of important additional confounders such as lipoprotein levels in more recent observational analyses of the obesity paradox [27]. We emphasise that our study, however, cannot discount an additional and potentially important neurohormonal and metabolic role for adipose tissue in our observations. This merits further study.

A significant finding in the current study was the association between intrarenal inflammation and both pro-survival and pro-apoptotic signalling. Previous studies have shown that the changes in renal vascular and tubular homeostasis that we have observed in response to a high-fat diet in swine are dependent on proinflammatory redox signalling [28,29]. A key signalling node in this respect is NF-κB [29-31], a redox-sensitive proinflammatory transcription factor the expression of which was increased by a high-fat diet in the current study, as was the expression of proteins encoded by several NF-κB-dependent genes, iNOS, VEGF and Bcl2 [32]. In contrast, in the CPB pigs fed a normal diet, acute increases in NF-κB as well as iNOS and Bcl2 expression were associated with loss of endothelial homeostasis as well as increases in cleaved caspase 3, a marker of apoptosis. This was associated with more severe inflammation, as determined by ET-1 expression and serum IL-6 levels, and ATP depletion observed following CPB. These are potent drivers of tubular epithelial apoptosis and endothelial injury [33-35] that may modify NF-κB signalling, as evinced in this case by the suppression of VEGF [36], and may promote cell death. It is also noteworthy that apoptosis in CPB pigs fed a normal diet was associated with activation of the key pro-survival node AKt. As with NF-κB it may be that the severity of the inflammation and ATP depletion caused by the CPB injury exceeded the protective effects of Akt signalling. In complete contrast, the preconditioning against AKI by a high-fat diet was independent of the phosphorylation of Akt, which is recognised as an important signalling node in acute ischaemic preconditioning [37]. Akt phosphorylation was in fact reduced in CPB + HF pigs relative to other groups, as was the expression of proteins in several related pro-survival signalling pathways including p70S6K (mTOR), Bcl2 (anti-apoptotic) and HIF-1α (angiogenesis). This is not entirely counterintuitive; decreased mammalian target of rapamycin signalling increases autophagy, an important protective mechanism in AKI [38]. Moreover, reduced mammalian target of rapamycin signalling alongside reduced HIF-1α expression promotes the polarisation of macrophages from the classically activated (M1) proinflammatory phenotype (thereby reducing IL-6 release) towards the alternatively activated reparative (M2) phenotype [39] that can promote renal recovery.

Of immediate translational relevance was our finding that inflammation was associated with both pro-survival and pro-apoptotic processes. More specifically, we observed pleiotropic effects of NF-κB signalling in the current study that have also been demonstrated in other models of renal vascular endothelial and tubular epithelial injury [40,41]. This suggests that inflammatory redox signalling can have a protective role in AKI, either as a form of preconditioning or perhaps as an important process in cell repair. Consequently, blanket inhibition of inflammation or oxidative stress may not confer an overall benefit to patients at risk of AKI, and this is supported by the failure of anti-inflammatory interventions such as statins, corticosteroids, and antioxidants to reduce post-cardiac surgery AKI severity in randomised trials [42,43]. Here we speculate that it is perhaps the dysregulation of inflammatory responses, typical of risk factors such as diabetes and chronic kidney disease [44,45], or prolonged CPB [3] that predisposes to clinical AKI.

## Conclusion

Our study demonstrates that high-fat feeding in swine results in obesity, hyperlipidaemia and renal inflammation. This protects against post-CPB AKI. Moreover, proinflammatory signalling was associated with both renal injury and protection. These observations demonstrate the complexity and redundancy of proinflammatory and pro-survival signalling pathways in the setting of post-CPB AKI, cast light on the failure of historical interventions to improve outcomes in patients at risk, and highlight the value of complex large animal recovery models in translational research.

## Key messages

• Observational analyses describe an association between obesity and improved clinical outcomes in the critically ill and those undergoing major surgery; a phenomenon that has been labelled the Obesity Paradox.

• No mechanism has been put forward to explain the Obesity Paradox, and the validity of these observations has been questioned.

• In this study we document that a high-fat diet, which results in hyperlipidaemia and obesity, protects against post-CPB AKI in swine by promoting renal inflammation, cellular proliferation and vascular homeostasis.

• This suggests that pre-existing inflammation may pre-condition organs against ischaemia reperfusion injury and provides a hypothetical basis for the Obesity Paradox observed in clinical studies.

## Abbreviations

AKI: Acute kidney injury; AKT: Serine/threonine-specific protein kinase (protein kinase B); Bcl2: B-cell lymphoma 2; CPB: Cardiopulmonary bypass; ET-1: Endothelin-1; FiO2: Fraction of inspired oxygen; GFR: Glomerular filtration rate; HIF-1α: Hypoxia inducible factor-1 alpha; IL: Interleukin; iNOS: Inducible nitric oxide synthase; NF-κB: P65 nuclear factor-kappa B; NGAL: Neutrophil gelatinase-associated lipocalcin; p70s6k: P70 ribosomal S6-kinase; PaO2: Partial pressure of oxygen; VEGF: Vascular endothelial growth factor.

## Competing interests

GJM has received consultancy fees from Abbott Pharmaceuticals in relation to AKI. The remaining authors declare that they have no competing interests.

## Authors’ contributions

PS developed the obesity model, undertook the *in vivo* experiments, the histological immunofluorescence analyses, and western blot analyses, and assisted with the interpretation of the data and the drafting of the manuscript. NNP developed the CPB model, undertook the *in vivo* experiments and assisted with the interpretation of the data and the drafting of the manuscript. HL undertook the reverse-phase high-performance liquid chromatography and immunohistochemical analyses, analysed the data and assisted with their interpretation. GJW undertook the enzyme-linked immunosorbent assay analyses and assisted with the statistical analyses and the drafting of the manuscript. PR developed the *in vivo* model, undertook the *in vivo* experiments and assisted with the analysis and interpretation of the clinical data. GIW and SCS helped design the study, supervised the histological and western blot analyses, and helped interpret the data and draft the manuscript. GJM conceived and designed the study, analysed and interpreted the data and drafted the manuscript. All authors read and approved the final manuscript.
